# Sweeps in time: leveraging the joint distribution of branch lengths

**DOI:** 10.1093/genetics/iyab119

**Published:** 2021-08-03

**Authors:** Gertjan Bisschop, Konrad Lohse, Derek Setter

**Affiliations:** Institute of Evolutionary Biology, University of Edinburgh, Edinburgh EH9 3FL, UK

**Keywords:** selective sweeps, positive selection, genealogy, inference, coalescent

## Abstract

Current methods of identifying positively selected regions in the genome are limited in two key ways: the underlying models cannot account for the timing of adaptive events and the comparison between models of selective sweeps and sequence data is generally made via simple summaries of genetic diversity. Here, we develop a tractable method of describing the effect of positive selection on the genealogical histories in the surrounding genome, explicitly modeling both the timing and context of an adaptive event. In addition, our framework allows us to go beyond analyzing polymorphism data via the site frequency spectrum or summaries thereof and instead leverage information contained in patterns of linked variants. Tests on both simulations and a human data example, as well as a comparison to SweepFinder2, show that even with very small sample sizes, our analytic framework has higher power to identify old selective sweeps and to correctly infer both the time and strength of selection. Finally, we derived the marginal distribution of genealogical branch lengths at a locus affected by selection acting at a linked site. This provides a much-needed link between our analytic understanding of the effects of sweeps on sequence variation and recent advances in simulation and heuristic inference procedures that allow researchers to examine the sequence of genealogical histories along the genome.

## Introduction

The variation we observe in genome sequence data is the result of the combined demographic and selective forces acting in the evolutionary history of a population. While demography shapes genetic variation uniformly throughout the genome, natural selection has localized effects on genetic variation near the targets of past selection. Recombination attenuates the strength of this effect with increasing distances from any selected site (Maynard Smith and Haigh 1974). Despite this key difference, distinguishing the signatures of natural selection from those of demography in genomic variation remains a significant challenge.

Substantial effort has been made to describe the effect of positive selection on the genealogical history at linked neutral sites and to develop methods to detect the footprint of adaptive evolution in genomic data [for an overview, see Hejase *et al.* (2020a)]. Here, we focus on the class of parametric model-based methods that identify the signature of hard selective sweeps as a local distortion of ancestry caused by genetic hitchhiking [for a survey of such methods, see Pavlidis and Alachiotis (2017)]. When a new adaptive mutation sweeps through a population, the hitchhiking of linked neutral variation leads to a local reduction in genetic diversity (Maynard Smith and Haigh 1974) and generates a statistically detectable footprint in the site frequency spectrum (SFS; Kim and Stephan 2002). This forms the basis for a number of composite likelihood methods to detect selective sweeps such as SweepFinder (Nielsen *et al.* 2005), SweepFinder2 (DeGiorgio *et al.* 2016), SweeD (Pavlidis *et al.* 2013), and for adaptive introgression sweeps, VolcanoFinder (Setter *et al.* 2020).

However, many of these methods are limited in at least three fundamental ways. Firstly, their focus on summaries of average diversity and divergence discards relevant information in the co-occurrence of closely linked variants. Secondly, assuming equilibrium population dynamics has been shown to increase both false-positive and false-negative error rates (Crisci *et al.* 2013). Finally, current sweep-scanning approaches assume that the population has been sampled at the time of fixation of the beneficial mutation, leading to a decrease in power to detect increasingly old sweeps. Given these limitations, it remains an open question how much additional information about past selective sweeps is contained in sequence variation.

### Approximating sweeps

Since the introduction of the hitchhiking model (Maynard Smith and Haigh 1974), many approximations for the effect of a selective sweep have been developed using the coalescent framework of Kingman (1982), Hudson (1983), and Tajima (1983). Here, the fixation of a new beneficial mutation has the effect of genetically structuring the ancestry at linked neutral loci (Kaplan *et al.* 1989; Stephan *et al.* 1992; Barton *et al.* 2004). During the sweep, coalescence can only occur among lineages on the same genetic background as the selected locus, while recombination may move lineages from the selected onto a neutral genetic background. However, analytic expressions to quantify these effects are only possible with further simplifications of the model. The genome scanning methods mentioned above are based on the star-like approximation for the selective sweep (Barton 1998, 2000; Durrett and Schweinsberg 2005; Berg and Coop 2015), which is relatively accurate, yet computationally tractable for thousands of samples (Pavlidis *et al.* 2013). We can view the star-like approximation as follows: assuming selection is strong ($N_{e}s\gg 1$), fixation of the beneficial mutation happens nearly instantaneously on the coalescent time scale. Lineages either recombine out of the sweep individually or coalesce in a single multiple-merger event at the origin of the beneficial mutation. However, this assumption fails either when selection is weak or at intermediate recombination distances from the selective target when selection is strong, and this leads to biased parameter estimates (Barton 1998; Santiago and Caballero 2005; Hartfield and Bataillon 2020; Setter *et al.* 2020; Charlesworth 2020). More accurate approximations for the effect of selective sweeps on genealogies have been developed (Bossert and Pfaffelhuber 2013), which, although more cumbersome mathematically, may avoid biases in parameter estimates and genome scans. The initial growth of a beneficial mutation behaves like a supercritical branching process (Kaplan *et al.* 1989; Evans and O’Connell 1994; Barton 1998). Conditioned on fixation, the stochastic increase in frequency is well-approximated by a pure-birth or Yule process. The structured coalescent that describes the genealogy at a linked neutral locus is then well-approximated by marking the lineages in the Yule tree by recombination events (Schweinsberg and Durrett 2005; Etheridge *et al.* 2006; Pfaffelhuber *et al.* 2006). Thus, in contrast to the star-like approximation, lineages on the selected background are assumed to coalesce pairwise during the sweep and can later recombine out of the sweep. Modeling and simulating the sweep phase as a time interval during which the coalescent is governed by the Yule process (Hermisson and Pfaffelhuber 2008) is possible for reasonably strong selection; however, analytic results are possible only for a sample of two. We will refer to this as the full Yule approximation. An alternative approach, which extends to larger samples (tens of individuals), is to use the Yule process to derive better approximations for a model that assumes that the sweep is instantaneous (on the coalescent time scale; Etheridge *et al.* 2006; Bossert and Pfaffelhuber 2013). The sampling formulae derived by Bossert and Pfaffelhuber (2013) assume that a sweep partitions lineages at a linked neutral locus into three families: nonrecombining lineages, early recombining lineages, and late recombinant lineages. We will refer to this as the instantaneous Yule approximation. Like the star-like approximation, the instantaneous Yule approximation is an instantaneous partitioning of the sample, but it allows for up to two multiple-merger events (Pfaffelhuber *et al.* 2006).

### Overview

The motivation of the present study is to develop a full analytic description of the effect of a hard selective sweep that occurred at an arbitrary time in the past on the distribution of genealogies at nearby neutral sites and to explore how this can be used to improve likelihood-based inference. We use forwards simulations throughout to quantify the robustness and accuracy of our analytic predictions and to assess the power of our method.

The paper is structured as follows: First, we briefly summarize approximate models of selective sweeps and show how a hard selective sweep occurring at an arbitrary time in the past can be embedded in the generating function (GF) for the distribution of the genealogy introduced by Lohse *et al.* (2011). The GF provides a recursive description of the full genealogy of a sample for a general class of structured coalescent processes with discrete events. While previous applications of the GF have focused on models of demographic history (Bunnefeld *et al.* 2015; Lohse *et al.* 2016), here, we use the GF framework to describe the genealogy at a neutral locus associated with a hard sweep occurring at a given time in the past.

Secondly, we use the GF to derive (and rederive) analytic predictions for the effect of a sweep on mean genetic diversity, the SFS, and the probability of genealogical topologies in the vicinity of a sweep target. In addition, we obtain the marginal distributions of the length of branches with *i* descendants among the sample (i-Ton branches) that underlie the SFS, and we compute the probability distribution of blockwise configurations of completely linked mutations (the blockwise SFS or bSFS) in the region of the genome affected by the selective sweep.

Finally, to connect these results to sequence data, we develop a simple composite likelihood framework based on the bSFS and assess the power and accuracy of our method to jointly estimate the sweep time and the strength of selection, comparing the performance of our method to that of SweepFinder2 (DeGiorgio *et al.* 2016). We also apply our method to the known sweep of the *C/T(-13910)* (rs4988235) mutation of the *MCM6* gene that underlies lactase persistence in European populations and discuss the bioinformatic challenges faced when using blockwise data for inference.

## Materials and methods

### Evolutionary history

**Figure 1:**
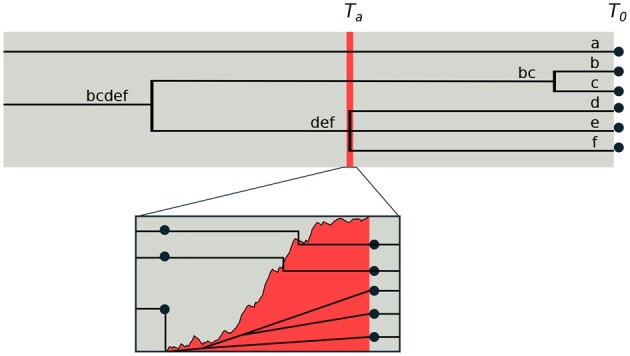
Model. The effect of an old selective sweep at time *T_a_* on a sample of six lineages {a,b,c,d,e,f} at a nearby neutral site. Tracing the genealogy pastward, we first observe a neutral coalescence of the *b* and *c* lineages. The second event is the selective sweep, which occurs quickly on the time scale of coalescent events. This induces what appears to be a multiple-merger coalescence of *d*, *e*, and *f* (as in the star-like approximation). On closer inspection, we see the stochastic frequency trajectory of the adaptive mutation (shown in red) that structures the coalescent during the sweep. Here, the *a* and *bc* lineages recombine out of the sweep, and although the events occur in rapid succession, the remaining lineages do indeed coalesce pairwise. Prior to the sweep, neutral coalescence of the remaining lineages continues until a common ancestor is found.

We consider *n* lineages sampled from a panmictic population of *N_e_* diploid individuals that evolves according to a Wright–Fisher model. We initially assume that each lineage is uniquely labeled, *i.e.*, the data are polarized relative to an outgroup and each haplotype is fully phased (we relax these assumptions when considering inference). In Figure 1, we uniquely label the lineages ancestral to each sampled individual a,b,c,d,e, and *f*. A coalescence event may then generate, *e.g.*, branch type *bc* which is ancestral to lineages *b* and* c*.

We measure time pastward from sampling ($T_{0}=0$) in units of $2N_{e}$ generations, *i.e.*, on the coalescent time scale. We consider a single selective sweep of a *de novo* beneficial (and codominant) mutation with selection coefficient *s* that swept to fixation at a discrete time point *T_a_* in the past. We define *T_a_* as the time interval between fixation of the beneficial mutation and the time of sampling so that $T_{a}\geq T_{0}=0$.

In the full model, the beneficial mutation sweeps through the population following a stochastic frequency trajectory X[t] satisfying X[T]=1 for $T\leq T_{a}$ and for some $T\prime >T_{a},\;X[T\prime ]=\frac{1}{2N_{e}}$ and X[T>T′]=0. That is, the beneficial mutation arises as a single new mutation in a randomly chosen background at time T′.

This frequency trajectory structures the coalescent process at linked neutral sites (Durrett and Schweinsberg 2004). Coalescence occurs only between lineages that share the state at the selected site. Lineage pairs currently associated with the beneficial (conversely, ancestral) allele may coalesce at rate $\frac{1}{2N_{e}X[t]}$ (respectively, $\frac{1}{2N_{e}(1-X[t])}$), while any single such lineages may recombine out of (*i.e.*, into, forwards in time) the sweep at rate r(1−X[t]) per generation (respectively, rX[t]). Here, *r* is the rate of recombination between the selected and the neutral site.

#### Describing the coalescent using GFs

The ancestry of a sample can be described by the pairwise coalescence of lineages until the common ancestor is reached (Kingman 1982; Hudson 1983; Tajima 1983), and the time between these events is exponentially distributed. Any two lineages coalesce independently at rate 1 (in units of $2N_{e}$ generations), so when *k* lineages remain in our sample, the waiting time to the next coalescent event is ($\begin{array}{c} k\\ 2 \end{array}$). Each next step is conditionally independent of the last, so the total time it takes to reach the common ancestor *t_mrca_* is distributed as the sum of the interevent time distributions of the process. Deriving the distribution of *t_mrca_* directly is not trivial and requires repeated integration, but it is easy to obtain if we describe the process using a GF. By using GFs, the distribution of the sum of independent random variables simply becomes the product of their respective GFs. By describing the distribution of random variables as a sum or integral transform, GFs provide a useful analytical tool for understanding random variables. In the GF, each variable in the time domain (*t_x_*) is associated with a corresponding “dummy” variable (*ω_x_*) in the new domain of the transform.

The GF approach as described in Lohse *et al.* (2011) uses the Laplace transform of the interevent times in the coalescent history. The Laplace transform is a natural choice and has a simple interpretation: it is the probability that the associated random event happens before the occurrence of some *catastrophe* with an exponentially distributed waiting time (Råde 1972). In coalescent terms, we can interpret the catastrophe as mutations occurring along the branches in the genealogy. Therefore, the Laplace transform of the distribution of genealogical branches is itself the probability of not seeing any mutations along the genealogy, *i.e.*, the probability of identity in state for the sampled lineages (Lohse *et al.* 2011). Associating each ancestral branch type with a unique dummy variable gives the GF for the distribution of all branch types. Because of its simple form, the GF can be obtained through a straightforward recursion that accounts for all possible sequences of events (and thereby, all possible topologies). Taking the inverse Laplace transform with respect to any particular set of dummy variables, we recover the joint probability distribution of the corresponding branch lengths.

Following the notations of Lohse *et al.* (2011), Hermisson and Pfaffelhuber (2008), and Barton *et al.* (2004), we label a sample of *n* lineages as a set {a,b,c…} and define the coalescence of the sample as a process that takes values in the set of partitions of {a,b,c…}. The process starts with the set of sampled lineages Ω={{a},{b},{c}…} and ends when all lineages coalesce, Ω={{a,b,c,…}}.

When describing the neutral coalescent, each term in the GF will consist of *n–*1 factors, each corresponding to the coalescence of two distinct lineages. In our set-notation, we represent each such coalescent event by the removal of lineages *i* and* j* from the indexed set Ω and replacing them with a single lineage representing their common ancestor, giving rise to a set $\Omega_{i,j}$ with $|\Omega_{i,j}|=|\Omega |-1$. The function Φ mathematically describes all possible events given a set of lineages, allowing us to define the recursion to obtain the neutral GF as

(1) $$\begin{array}{cc} \Phi [\Omega ] & :=\frac{1}{\left(\begin{array}{c} \left|\Omega \right|\\ 2 \end{array}\right)+\sum_{x\in \Omega }\omega_{x}}\cdot \sum_{1\leq i<j\leq |\Omega |}\Phi [\Omega_{i,j}] \end{array},$$

where the sum over 1≤i<j≤|Ω| represents the set of possible pairwise coalescent events among the Ω lineages and the dummy variable terms *ω_x_* are summed over all lineages *x* present in Ω. When |Ω|=1, Φ[Ω]=1 and the recursion ends.

#### Embedding sweeps in the Kingman coalescent

For times $T<T_{a}$, *i.e.*, more recently than the selective sweep occurs, the ancestry of the sample Ω is described by the Kingman coalescent (Kingman 1982).

Although the sweep is a discrete event, following Lohse *et al.* (2011), we initially treat the sweep as a competing exponential process occurring at rate *δ* backward in time. This allows us to obtain through recursion the GF for the distribution of branch lengths in the genealogical history. By taking the inverse Laplace transform of the GF divided by *δ*, we recover the GF parameterized by the discrete time when the beneficial mutation reaches fixation *T_a_*.

(2) $$\begin{array}{cc} \Phi [\Omega ,\delta ] & :=\frac{1}{\left(\begin{array}{c} \left|\Omega \right|\\ 2 \end{array}\right)+\delta +\sum_{x\in \Omega }\omega_{x}}\\ & \cdot (\sum_{1\leq i<j\leq |\Omega |}\Phi [\Omega_{i,j},\delta ]+\delta *\Phi^{*}[\Omega ]) \end{array}$$

$\Phi^{*}$ represents the recursive term for the effect of the adaptive event on the genealogy of our sample. Throughout the paper, we use the superscript ${}^{*}$ to distinguish functions corresponding to models of selection from those of the neutral model, *i.e.*, those without a superscript. Here, we focus on two different instantaneous sweep approximations: the star-like approximation and the instantaneous Yule approximation. Both of these approximations describe the impact of a sweep as a partitioning of the extant lineages. As such, these instantaneous events do not add length to any of the branches and thus $\Phi^{*}[\Omega ]=\Phi [\Omega \prime ]$ (see equation 1) with Ω′ a partition of Ω as described by either approximation. Note that $\Phi^{*}$ no longer depends on *δ*, so that the sweep may only occur once in the genealogical history. Also note that each term of the GF will now contain at most *n* factors.

#### The star-like approximation

In the star-like approximation, the neutral lineages sampled at a locus *d* bases from the adaptive mutation independently recombine out of the sweep (*i.e.*, they *escape*) with probability $P_{e}=1-e^{-\alpha }$. The parameter *α* measures the strength of the sweep relative to the total rate of recombination between the neutral and selected site: $\alpha =\frac{r*d}{s}\ln[2N_{e}s]$, where *r* is the per-base recombination rate, *N_e_* is the (diploid) population size, and *s* is the heterozygous advantage of the beneficial mutation. From another perspective, the duration of a selective sweep (the time to fixation) is approximately $t_{\mathrm{fix}}=2\ln[2N_{e}s]/s$ (in generations), and the probability that no recombination occurs during this interval, $e^{-\alpha }$, depends on the total rate of recombination during the sweep through $\alpha =r*d*t_{\mathrm{fix}}/2$. The nonrecombinant lineages that do not “escape” the sweep coalesce instantaneously to the origin of the beneficial mutation (Barton 1998, 2000; Durrett and Schweinsberg 2005). This approximation thus partitions *n* extant lineages into two sets, either of which may be empty, with one representing the set of *m* lineages that escape the sweep and the other the multiple merger of the *n–m* remaining lineages . The probability of observing such an event will be denoted by $P_{m,n}$, *i.e.*, the probability that *m* out of *n* lineages escape the sweep.

#### The instantaneous Yule approximation

This sampling formula considers a partition into three sets and is based on the Yule approximation (Etheridge *et al.* 2006; Bossert and Pfaffelhuber 2013). Provided $2N_{e}s$ is sufficiently large, the trajectory X[t] of a beneficial mutation under (strong) selection can be more closely approximated by considering a pure-birth process with binary splits at rate $2N_{e}s$ (Schweinsberg and Durrett 2005). Forward in time, this Yule process describes the ancestry of all lineages descending from the beneficial mutation present at the end of the sweep (*i.e.*, those with an infinite line of descent). Note that this process is stopped once there are $4N_{e}s$ lineages, given that each lineage has a probability 2*s* of having an infinite line of descent. Genealogies under hitchhiking at neutral sites, at a recombination distance r*d from the sweep site, can then be described by marking the Yule tree along its branches with recombination events occurring at rate $2N_{e}*r*d$. Now, letting Ω represent the set of lineages present at time *T_a_*, we can define a labeled partition induced by the Yule process that governs the coalescent during the sweep. This partition consists of three families:

1.
|L|=l late recombinant singletons: single lineages that have recombined away from the beneficial background.
2. A single family of early recombinants of size |E|=e: a family of lineages that recombines away after coalescing.
3. A single nonrecombinant family of size |N|=|Ω|−|L|−|E|: a family of lineages that is identical by descent to the founder of the sweep (along a distance of at least *d*).

### Simulations

The full model is implemented as a Wright–Fisher simulation using SLiM3.3 (Haller and Messer 2019) and msprime (Kelleher *et al.* 2016). Samples are extracted at a fixed number of generations after the sweep completes. Sequences are always 1 Mb in length, with the site under selection in the center. We assume a population with $N_{e}=10,000,\;r=1e^{-7}$ and $\mu =1.25e^{-7}$ throughout, and simulate samples of varying size (n∈[4,12,20]), sweep times ($T_{a}\in [0.1,0.5,1.0,2.0]$), and two different strength of selection (*s *=* *0.05 or 0.005).

### Power analysis

We assess the power to identify sweeps and the accuracy to infer sweep parameters (*T_a _*and* s*) using a composite likelihood (*CL*) scheme based on the bSFS [see Lohse *et al.* (2016) and *Results*]. Neutral variation for each of the B/2 blocks of fixed length *l* on either side of a putative sweep target can be summarized as a vector *k* by counting the mutation types occurring in that block. By taking derivatives of the GF with respect to the corresponding dummy variables *ω_x_* as derived in equation (30) of Bahlo and Griffiths (2001), probabilities for all vectors *k* can be obtained. Blocks immediately to the right and left of the sweep target have an average distance l/2. Although we may sample a larger number of individuals *n*, analytic results for the bSFS are limited to smaller sample sizes. In the *CL* framework, we accommodate this by considering all possible subsamples of size *x* (we use *x *=* *4 throughout). Let $P[k_{ij}]$ be the probability of observing a blockwise mutation configuration *k* at distance i*l−l/2 in subsample j, $1\leq j\leq \left(\begin{array}{c} n\\ x \end{array}\right)$. Summing over all $\left(\begin{array}{c} n\\ x \end{array}\right)$ subsamples of *n*, we can define the following *CL* for the sweep model,

(3) $$\mathrm{lnC}L_{s}(\theta ,T_{a},s)=\sum_{i=1}^{B}\sum_{j=1}^{\left(\begin{array}{c} n\\ x \end{array}\right)}lnP[k_{ij}].$$

Given an analogous likelihood under neutrality $\mathrm{lnC}L_{0}(\theta )$ the support for a sweep (at time *T_a_* and of strength *s*) can be measured as:

(4) $$\Delta \mathrm{lnCL}=\mathrm{lnC}L_{s}(\theta ,T_{a},s)-\mathrm{lnC}L_{0}(\theta ).$$

We fit both models to 1000 simulated replicates with a beneficial mutation as well as to 10,000 neutral simulations. To allow comparison, we repeat the analysis on the same data using SweepFinder2 (DeGiorgio *et al.* 2016). To measure power, we construct ROC curves: ΔlnCL values for *true* (hard sweep) and *false* (neutral) replicates are jointly ranked in descending order, after which, for each element, both the fraction of false and true positives are determined. Note that we do not perform a sweep scan but rather assume that the position of the selective target is known.

Rather than evaluating all equations for each combination of parameter values ($\theta ,Ta,s,N_{e},r$), we construct an interpolation function (third-degree polynomial) for each mutation configuration, from a grid of pre-evaluated mutation configuration probabilities in *Mathematica* (version 12). Evaluating a polynomial rather than the exact analytical expression reduces computation time significantly. For each replicate, inference consists of two steps: we first estimate *θ*, using all blocks that are sufficiently far away from the sweep site (α>12). We then obtain joint estimates of *Ta* and* s* conditional on *θ*. Parameter optimization is conducted on a grid (θ,Ta,s) allowing us to precompute all bSFS configurations, and run the optimization for all simulation replicates on a laptop.

## Results

### Time erodes the footprint of adaptive evolution

In this section, we examine how the expected footprint of adaptive evolution is affected by *T_a_*, the time since the selective sweep. We first show results for pairwise genetic diversity (*n *=* *2) and then extend this to the SFS (*n *=* *9). A detailed analysis as well as an illustration of our approach is provided in Supplementary S1 Notebook. Note that throughout we use the superscript ${}^{*}$ to denote GFs and distributions for coalescent histories with a sweep event.

#### Pairwise genetic diversity

For illustration, we first derive the GF for the distribution of branch lengths in a sample of two (haploid) lineages. In this case, the two branches *a* and* b* are equivalent, and their sum is twice the time to the most recent common ancestor ($t_{\mathrm{mrca}})$. By substituting $\omega_{a}+\omega_{b}\to \omega_{\mathrm{mrca}}=\omega$, we obtain the GF for *t_mrca_*. Under the neutral model, the GF is simply the Laplace transform of an exponentially distributed random variable with mean 1

$$\phi =\frac{1}{1+\omega }.$$

Using the star-like approximation of a selective sweep, the recursion for the GF with parameter *δ* results in:

$$\begin{array}{cc} \phi^{*}[\omega ,\delta ] & =\frac{1}{1+\delta +\omega }+\frac{\delta P_{0,2}}{1+\delta +\omega }\\ & +\frac{\delta P_{1,2}}{1+\delta +\omega }\cdot \frac{1}{1+\omega }+\frac{\delta P_{2,2}}{1+\delta +\omega }\cdot \frac{1}{1+\omega }. \end{array}$$

The summed terms represent the possible sequences of events in the genealogical history of the sample and $P_{i,j}$ represents the probability that *i* out of *j* lineages escape the sweep. The first term corresponds to neutral coalescence before the sweep; the second, to coalescence during the sweep. In the remaining terms, one or both lineages escape the sweep and subsequently coalesce under the standard neutral coalescent. Since we have defined the GF for a model with exponentially distributed sweep times $\phi^{*}[\omega ,\delta ]=\int_{0}^{\infty }\delta e^{-T_{a}\delta }\phi^{*}[\omega ,T_{a}]dT_{a}$. Taking the inverse Laplace transform of $\phi^{*}[\omega ,\delta ]/\delta$ gives us the GF for the distribution of branch lengths as a function of the time since the sweep *T_a_*:

$$\begin{array}{cc} \phi^{*}[\omega ,T_{a}] & =e^{-T_{a}(1+\omega )}P_{0,2}\\ & +\frac{1}{1+\omega }*\left(\left(1-e^{-T_{a}(1+\omega )})+e^{-T_{a}(1+\omega )}(1-P_{0,2}\right)\right). \end{array}$$

In the limit as $\omega \to 0,\;\phi^{*}$ becomes a sum of terms, each representing the probability of a particular genealogical history (Lohse *et al.* 2011). Neutral coalescence occurs before the sweep with probability $1-e^{-T_{a}}$. Given that it does not (with prob. $e^{-T_{a}}$), coalescence may happen during the sweep with probability $P_{0,2}$, or neutrally after the sweep with probability $\left(1-P_{0,2}\right)$. The expected time to the most recent common ancestor $E[t_{\mathrm{mrca}}]$ is obtained by taking minus the derivative of the GF with respect to *ω* and then taking the limit as ω→0 (Lohse *et al.* 2011). In the neutral case, $E[t_{\mathrm{mrca}}]=1$, and for the sweep scenario, substituting $P_{0,2}=e^{-2\alpha },\;E[t_{\mathrm{mrca}}]=1-e^{-T_{a}}P_{0,2}=1-e^{-T_{a}-2\alpha }$.

**Figure 2:**
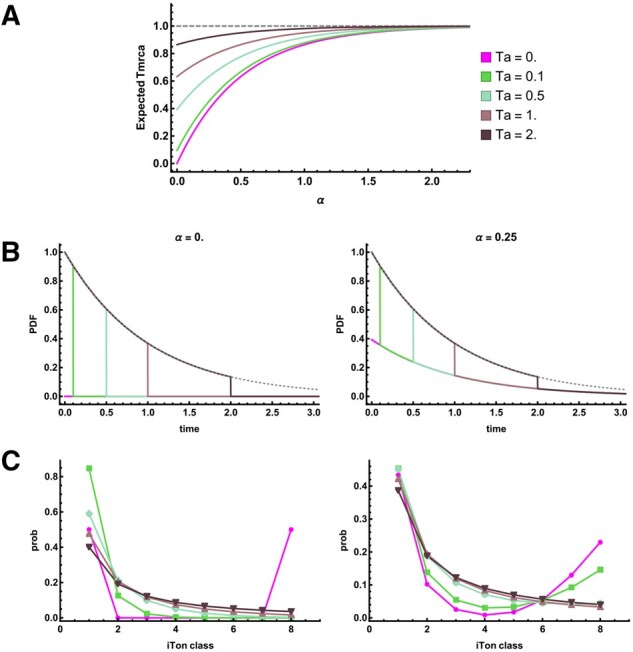
The signature of old sweeps, star-like approximation. (A) The effect of a sweep on the expected time to the most recent common ancestor (*t_mrca_*) as a function of the distance from the sweep center ($\alpha =\frac{r}{s}\ln[2N_{e}s]$) and the time since the sweep *T_a_*. (B) The distribution (PDF) of *t_mrca_* at the sweep center, *α*  =  0 and at distance α=0.25. (C) The SFS for a sample of *n *=* *9 individuals.

When *T_a_* = 0, *i.e.*, the population is sampled at the time of fixation, we recover the classic valley of diversity caused by a selective sweep (Maynard Smith and Haigh 1974; Kaplan *et al.* 1989; Figure 2A). By comparison, older sweeps have a reduced effect on $E[t_{\mathrm{mrca}}]$. Forwards in time, this amounts to the recovery of genetic diversity that was lost due to hitchhiking in the selective sweep. From a coalescent viewpoint, the genealogy is unaffected by selection if coalescence occurs before the sweep.

#### Distribution of *t_mrca_*

The effect old sweeps have on the genealogy can be seen more clearly in the full distribution of *t_mrca_*: under the neutral model, *t_mrca_* is exponentially distributed with rate 1. The probability density (PDF) and cumulative density functions (CDF) are therefore $f[t]=e^{-t}$ and $F[t]=1-e^{-t}$, respectively. Under the selection model, we obtain the PDF by inverting the GF with respect to *ω* (Lohse *et al.* 2011). We may integrate the PDF with respect to *t* to obtain the CDF or alternatively, we may divide the GF by *ω* and then take the inverse Laplace transform. For this model, we obtain the PDF ($f^{*}$) and CDF ($F^{*}$) for *t_mrca_* at a neutral locus linked to the adaptive mutation:

$$f^{*}[t]=\left\{\begin{array}{ll} e^{-t} & 0\leq t<T_{a}\\ e^{-t}(1-P_{0,2})+P_{0,2}e^{-Ta} & t=T_{a}\\ e^{-t}(1-P_{0,2}) & t>T_{a} \end{array}\right.$$

$$F^{*}[t]=\left\{\begin{array}{ll} 1-e^{-t} & t<T_{a}\\ 1-e^{-t}(1-P_{0,2}) & t\geq T_{a} \end{array}\right.$$

.

As expected, for times $t<T_{a}$, the PDF matches the neutral case, $f^{*}[t]=f[t]$, since the sweep cannot affect the genealogy during that period (Figure 2B). Since we assume that the sweep induces an instantaneous coalescent event, there is a point mass of size $e^{-T_{a}}P_{0,2}=e^{-T_{a}-2\alpha }$ at *t* = *T_a_*. Indeed, at the sweep center, all coalescence occurs before or during the sweep $t\leq T_{a}$. At greater distances from the sweep center, the point mass diminishes in size. For $t>T_{a}$, only lineages that escaped the sweep may subsequently coalesce, and they do so neutrally. Thus, the probability density matches the neutral case scaled by the probability that one or both lineages escape, $f^{*}[t]=e^{-t}(1-P_{0,2})=f[t](1-P_{0,2})$. Indeed, Figure 2 shows that, although the location of the discontinuity shifts as the time since the sweep increases, the probability density for $t>T_{a}$ is determined only by the distance from the sweep center, $\alpha =\frac{r*d}{s}\ln[2N_{e}s]$.

#### The SFS

For moderate sample sizes, we can obtain the expected SFS as a function of both the distance from the sweep center *α* and* T_a_* the time since the sweep. Distinguishing branches by the number of descendants, *e.g.*, $\omega_{a,b}\to \omega_{2}$, the set of *ω_i_*, 1≤i≤n−1 corresponds to the length of the branches with *i* descendants among the sample (i-Tons). The expected *marginal* lengths of i-Ton branches $E[t_{i}]$ can be obtained by differentiating the GF with respect to *ω_i_*, analogous to $E[t_{\mathrm{mrca}}]$ described above for *n *=* *2. Normalizing by $\sum_{i=1}^{n-1}E[t_{i}]$ yields the expected frequency of mutations belonging to each i-Ton class. Figure 2C shows the SFS for a sample of *n *=* *9, at different distances from the sweep center and for increasingly old sweeps. As for the expected pairwise genetic diversity, the effect of older sweeps on the SFS is dampened. However, the relative effect differs between i-Ton classes and depends on both *T_a _*and* α*. At the sweep center, *α* = 0, we observe a prominent excess in the proportion of singleton lineages. In contrast, outside the sweep center, α=0.25, we see an excess of both intermediate and high-frequency polymorphisms as the age of the sweep increases.

### Beyond the mean—leveraging joint branch length information

Pairwise and/or average measures of sequence variation such as the SFS are drastic summaries. In order to fully capture the footprint of selective sweeps on linked neutral sequence variation, we would ideally like to compute the probability of haplotypic variation flanking a selective target. Unfortunately, this requires including recombination (including breakpoint locations) explicitly in the GF recursion which quickly becomes intractable. In the following, we focus on blocks of nonrecombining sequence and consider the effect of sweeps on three quantities of interest: the probability of genealogical topologies, the marginal distribution of i-Ton branches, and, following Lohse *et al.* (2016), the bSFS, the blockwise configuration of i-Ton counts.

#### The probability of genealogical topologies

**Figure 3:**
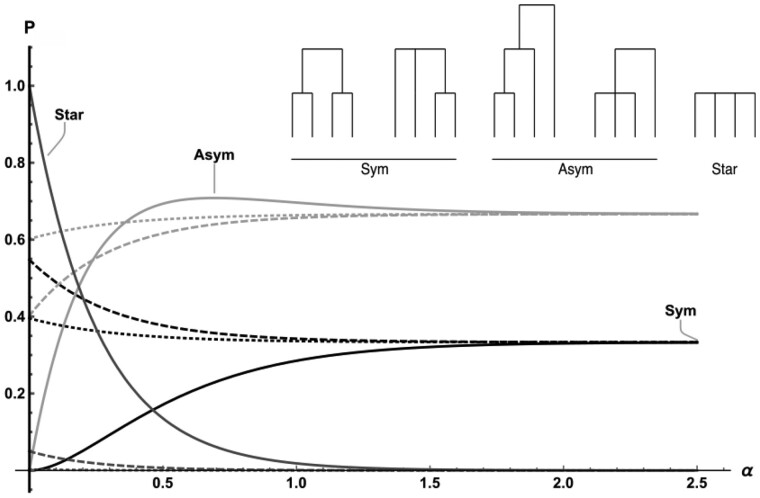
Probability of genealogical topologies for *n *=* *4, star-like approximation. The probability of a genealogy with an asymmetric (light gray), symmetric (black), or star-shaped (dark gray) root node is shown for *T_a_* = 0 (full), 0.5 (dashed), 1.0 (dotted), with increasing distance from the sweep center (left to right).

The probability of seeing any particular topology can be found by evaluating the limit at infinity for the *ω* corresponding to branches that are incompatible with it (and evaluating all other *ω* at zero). Under the star-like approximation and for *n *=* *4, this results in five different topologies, three of which are induced by multiple mergers. For the sake of simplicity, we can distinguish between three topology classes defined by the root node: genealogies with a symmetric or asymmetric bipartition at the root and genealogies without any bi-partition (*P_star_*; Figure 3).

$$\begin{array}{l} P_{sym}=\frac{1}{3}(1-2e^{-6Ta}(1-e^{3Ta})*2P_{0,3}-e^{-6Ta}(P_{0,4}+P_{1,4}))\\ P_{asym}=\frac{1}{3}(2+2e^{-6Ta}(1-e^{3Ta})*2P_{0,3}-e^{-6Ta}(2P_{0,4}-P_{1,4}))\\ P_{star}=e^{-6Ta}P_{0,4}. \end{array}$$

Dissecting the three terms in *P_sym_ and P_asym_*, the first term represents the probability of seeing (a)symmetric trees under the standard neutral coalescent. Only multiple mergers of three (second term) or four (third term) lineages will affect this probability.

#### The marginal distribution of i-Ton branches

The marginal distribution (PDF) of i-Ton branches, *i.e.*, the genealogical branches underlying the SFS (Figure 2C), can be obtained by inverting the GF with respect to *ω_i_*. The resulting expressions are cumbersome and are provided in Supplementary S1 Notebook for a sample size of four, which we investigate below.

For the case of *n *=* *4 and assuming neutrality, only two topologies are possible: the first coalescence event always generates a doubleton lineage, while the second may either generate a second doubleton lineage, resulting in a symmetric topology with probability $P_{\mathrm{sym}}=1/3$, or a tripleton lineage, resulting in an asymmetric topology with probability $P_{\mathrm{asym}}=2/3$. Thus, the marginal PDF of tripleton branches $f[t_{3}]$ contains a point-mass at $t_{3}=0$ of size 1/3, while the PDF for *t*_1_ and *t*_2_ contain no discontinuities (Supplementary Figure S1A).

**Figure 4:**
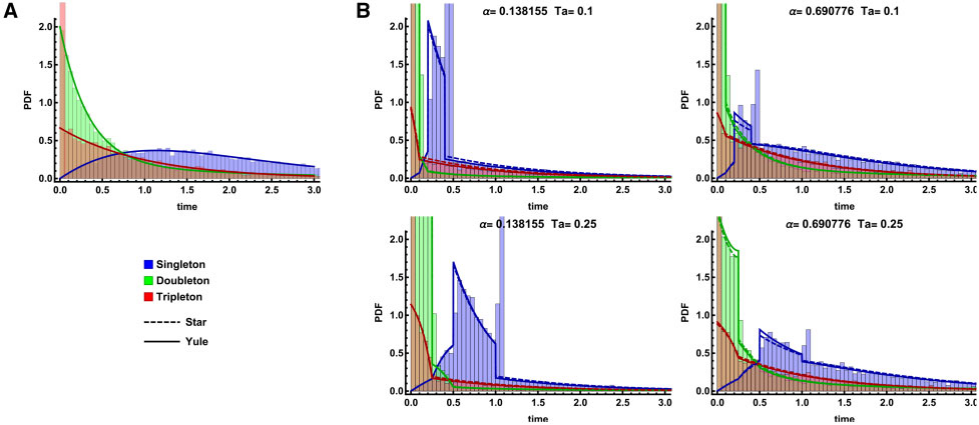
Marginal i-Ton branch length distributions for *n *=* *4. Analytic predictions under the neutral model (A) and the approximate selection models (B) are compared to the corresponding distribution obtained from 10,000 simulation replicates overlaid as a histogram. The Yule approximation is indicated by solid lines while the dashed lines indicate the star-like approximation. Results for singleton, doubleton, and tripleton branch lengths are shown in blue, green, and red, respectively. The top row shows two distances from the sweep center α≈{0.14,0.069} and $T_{a}=0.1$. Analogous results for an older sweep at $T_{a}=0.25$ are shown in the bottom row. Time is measured in units of $2N_{e}$ generations. Here, $N_{e}=10,000$, *s *=* *0.05, and $r=10^{-7}$. Note that the location and size of each point mass (*e.g.*, the tripleton point mass at time *t *=* *0) is reflected in the CDF rather than the PDF (Supplementary Figure S1).

In contrast, in the vicinity of a selective sweep, we observe multiple removable discontinuities in all three marginal PDFs (Figure 4). The PDFs can be rewritten as piece-wise continuous functions combining a continuous distribution of coalescence times with point masses corresponding to either the absence of a particular branch type or a burst of coalescence caused by the sweep. Given that each class of i-Ton branches consists of multiple genealogical branches, these distributions are more intricate than for the pairwise pairwise case ($f[t_{2}]$ above) and are discussed further in Supplementary S1 Notebook for the case of *n *=* *4.

In general, for a sample of size *n*, the discontinuities present in the branch length distribution of each i-Ton type are determined by the total number of such i-Ton branches present during the interevent times of the coalescent process during which the selective sweep occurs. For example, there are always *n* singleton branches present initially, and the first coalescent event reduces this to *n–*2. There exists one topology in which the number of singleton branches is reduced by one in each subsequent interval. Therefore, the PDF of singletons has a total of *n–*1 discontinuities at $t=\{(n)T_{a},(n-2)T_{a},(n-3)T_{a},\ldots ,T_{a}\}$. For *i *>* *1, there is always a point mass at *t *=* *0 due to the possibility that the first event is coalescence of all lineages during the sweep. The possible multiplicity of the (*i *>* *1)-Ton classes is determined by the ways to decompose *n* into smaller-valued integers and thus the number of discontinuities for *i *>* *1 is ⌊n/i⌋+1.

Finally, we note that the star-like approximation used for the analysis provides relatively accurate predictions. In comparison to simulations, the accuracy improves only slightly using the Yule approximation (Supplementary Figure S1). For both approximations, the model underestimates the time since the sweep occurred, *i.e.*, the location of the point-mass *T_a_*. In our model, we assume that the duration of the sweep (on the coalescent scale) $t_{\mathrm{fix}}/(2N_{e})$ is negligible. In reality, the burst of coalescence occurs at the beginning of the sweep, and including this extra time in our model (substituting $T_{a}+t_{\mathrm{fix}}$ for *T_a_*) largely accounts for the bias.

#### The bSFS

Above, we used the GF to obtain the SFS by deriving the expected length of i-Ton branches. An alternative and less drastic summary of sequence variation is the bSFS, the vector of SFS counts in short blocks (Bunnefeld *et al.* 2015). To be able to leverage topology information, we will focus on (sub)samples of *n *=* *4. In this case, the bSFS is a vector of counts for three i-Ton types k={k1,k2,k3} where $k_{i}\in \{0,1\ldots ,k_{\max}+1\}$. For example, a mutational configuration of (0, 0, 1) represents a block with only one tripleton mutation. Note that we use $k_{\max}+1$ to bin all mutation configurations with more than *k_max_* mutations of type *i*. If we restrict the bSFS to a maximum of *k_max_* = 2 mutations per i-Ton type, we distinguish ${\left(k_{\max}+2\right)}^{3}=64$ unique bSFS configurations (given that both the absence of a particular mutation type *k_i_* = 0 and seeing $>k_{\max}$ mutations also define bSFS configurations).

**Figure 5:**
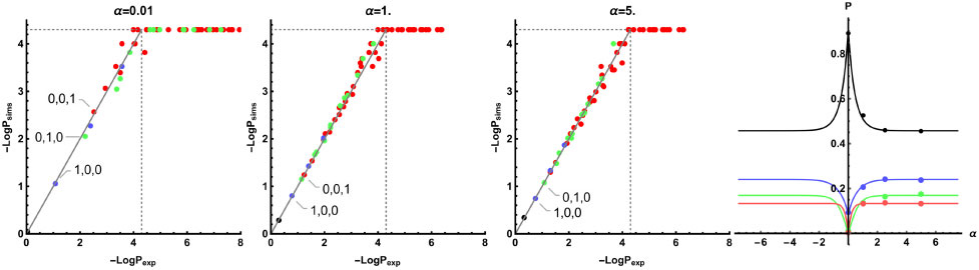
The bSFS for *n *=* *4 and $T_{a}=0.1$. The expected probabilities of bSFS configurations given by the star-like approximation (logscale) against their observed frequencies in 10,000 simulation replicates. Each dot corresponds to a unique bSFS configuration. Counts left and right of the selected site are added together. Each dot in the scatter plot represents a unique bSFS-configuration, counting the number of (singletons, doubletons, and tripletons). Red: (_,_,k), green: (_,k,0), blue: (k,0,0), black: (0,0,0) with k≥1, and _ any integer. The dotted line marks the minimal detectable frequency for the simulations. The rightmost figure shows the total probability of observing blocks within each of these categories (sweep center at α=0.0).

Assuming no recombination within blocks, the bSFS can be obtained from the GF by taking successive derivatives with respect to the *ω_i_* [see eq. (1) in Lohse *et al.* (2011) for details]. Comparing the analytic expectation for the bSFS $P[\underline{k}]$ to simulations (Figure 5) highlights both the accuracy of the star-like approximation (for *n *=* *4) and the robustness of the bSFS to intrablock recombination, provided blocks are short (here a recombination rate of $r=10^{-7}$, *l *=* *100 bases).

### Power to infer old sweeps

We can use the analytic result for the bSFS obtained above to jointly estimate the sweep time and the strength of selection in a *CL* framework (summing *lnL* across both blocks and subsamples of *x *=* *4, see *Materials and* *Methods*). In the following, we quantify the power (and bias) of characterizing sweeps using the star-like approximation and test to what extent the instantaneous Yule approximation improves these estimates.

**Figure 6:**
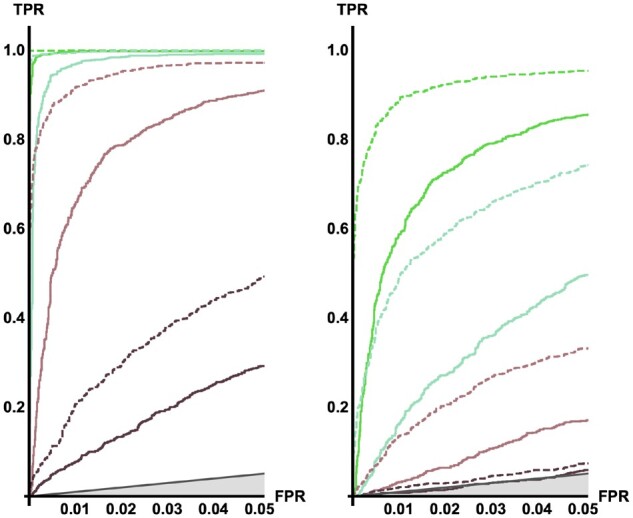
ROC curve, star-like approximation. Plotting the rate of true positives against the rate of false negatives shows how much power we have to distinguish genomic regions that underwent a hard sweep from neutral replicates. As expected, power depends on the time since the sweep [$T_{a}=0.1$ (green), 0.5 (lighter green), 1.0 (light brown), and 2.0 (dark brown)], the strength of selection (left *s *=* *0.05, right *s *=* *0.005) and sample size *n *=* *4 (full line), 12 (dashed).

**Figure 7:**
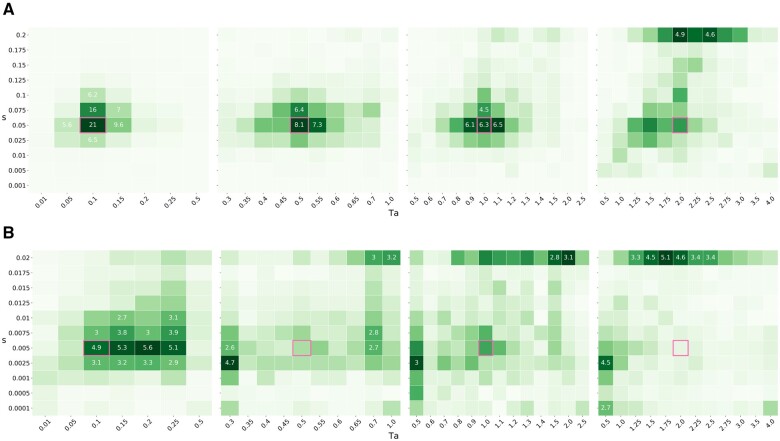
Heatmaps. Parameter estimates of the gridded optimization using the star-like approximation across simulations using a sample of *n* = 4 lineages. The top and bottom rows show strong and weak selection, respectively. The panels show the accuracy of our parameter estimates for simulation data with increasingly older sweeps ($T_{a}=0.1,0.5,1.0,2.0$ from left to right). Within the panels, each square represents a parameter combination in the test grid. The number inside each square shows the percentage of replicates (>4.5%) associated with a particular parameter combination, with darker shading corresponding to a higher density. The true simulated parameter combination is highlighted by a pink square. (A) Strong selection, *s* = 0.05 and (B) weak selection, *s* = 0.005.

With strong selection (*s *=* *0.05), the power to infer *T_a _*and* α* is high, even for fairly old sweeps ($T_{a}=1.0$), especially with samples of n≥12 (Figure 6). Even for small sample sizes (*n *=* *4), we get decent estimates of the sweep parameters (Figure 7). Increasing sample size (for a fixed subsample size of *x *=* *4) reduces mutational sampling noise but only increases power to estimate parameters to a limited extent (Supplementary Figure S2). The power to correctly infer sweep parameters decreases with increasing age of the sweep (*T_a_*). This is unsurprising, given that the number of lineages that enter the sweep, and hence the information about the sweep, declines with increasing *T_a_*.

When selection is weak (*s *=* *0.005), power drops off quickly for sweeps that are older than $T_{a}=0.5$, especially for small samples (*n *=* *4). The heatmap reveals that, irrespective of sample size, sweep parameters become nonidentifiable when selection is weak (Figure 7 and Supplementary Figure S3). Two effects are at play here: firstly, for weak selection, the assumption that sweeps happen instantaneously becomes problematic, as the duration of the sweep will be approximately 0.1. The fact that we estimate the time to the completion of the sweep using an approximation that assumes an instantaneous burst of coalescence at the onset will tend to bias estimates (towards higher *T_a_* values) when the duration of the sweep is on the same order of magnitude as the time since completion ($T_{a}\leq 0.5$). Secondly, when the model becomes nonidentifiable, we see the estimates for a fraction of the replicates veer off toward either larger *s* or smaller *T_a_*. Presumably, this is a consequence of the stochasticity of the coalescent which inherently limits the ability to detect a single weak sweep that affects only a small region of the genome. Depending on the particular realization of the neutral coalescent for the lineages remaining at this region, weak sweeps of intermediate age appear to be difficult to distinguish from much older and harder sweeps. We suspect that this is an inherent limitation of the signal in the data that cannot be overcome by adding more samples when the subsample size is kept small.

By contrasting ROC curves between a model with *T_a_* = 0 and a model where *T_a_* is free to vary, we can assess how much better the data fit an old sweep (Supplementary Figure S4). As expected, forcing *T_a_* = 0 (a standard assumption of sweep scans), works well for recent sweeps but breaks down for older ones, *i.e.*, power drops off in the same way previously reported for SFS-based methods (Racimo *et al.* 2014; Setter *et al.* 2020). However, when including the sweep time as a parameter, old sweeps become detectable with high power as long as they are strong.

Comparing the heatmaps and ROC curves under the star-like and the instantaneous Yule approximations (Supplementary Figures S5–S7) reveals very little difference between the two approximations in terms of accuracy and power. Root mean square errors for the estimates are nearly identical across a range of *T_a_* estimates. We also find that old sweeps are similarly nonidentifiable under both approximations when selection is weak suggesting that the power to infer selection is inherently limited in this case.

### Comparison to SweepFinder2

Analyzing the same set of simulations for SFS data using SweepFinder2, we clearly see that diagnosing sweeps using the bSFS has greater power across all parameter combinations (Supplementary Figures S8 and S10). SweepFinder2 only has power to detect strong sweeps when $T_{a}\leq 0.1$ and when n≥12. This increase in power is not only due to our ability to fit an additional parameter (Supplementary Figure S4). Using the bSFS clearly also allows us to extract more information from a limited number of samples (Supplementary Figure S9).

### Sweeps in the lactase gene region

We applied our method to estimate the timing and strength of selection acting on the *C/T(-13910)* (rs4988235) mutation in the MCM6 gene. This mutation, which is at high frequency in northern European populations (Enattah *et al.* 2002), is associated with lactose metabolism in adulthood (Järvelä 2005) and has strong support for a hard selective sweep (Bersaglieri *et al.* 2004; Coelho *et al.* 2005; Mathieson and Mathieson 2018; Speidel *et al.* 2019; Stern *et al.* 2019; Mathieson 2020). We used phase-3 data from the 1000 genomes project (The 1000 Genomes Project Consortium 2015), restricting our analysis to the European (CEU) samples and the 4-Mb region centered on the sweep target. Because the sweep is partial, we subsampled further, using only individuals that are homozygous for the causal variant. We obtained the bSFS for nonoverlapping 1000-bp blocks along the genome, including all sites but only considering variation at biallelic SNPs. The scaled mutation rate is estimated using the GF for a neutral coalescent history.

Analyzing bSFS variation 1 Mb on either side of the causal variant, we observe strong support for a hard selective sweep at rs4988235 with maximum *CL* estimates of *s *=* *0.086 and $T_{a}=0.0$ for the strength and timing of the sweep. This estimate of *s* is substantially higher than previous estimates. Inspection of test sites at 50-kb intervals in the flanking region of the genome (see Supplementary Figure S11) reveals apparently different signals on either side of the causal variant: while there is strong support for the neutral model (s→0 and $T_{a}>2$) upstream, a large (≈350kb) region downstream of rs498823 shows support for a selective sweep. These apparently conflicting results are an artifact of our simplifying assumption of a single estimated scaled mutation rate θ=0.44 (and selective neutrality) for the entire region, which ignores the fact that the downstream region is gene rich and so under strong selective constraint. Because the bSFS is highly sensitive to the scaled mutation rate, the neutral model poorly fits the data in this region. However, a model of strong positive selection can at least partially account for the low diversity observed, leading to inflated likelihood ratio scores. If we limit estimation of sweep parameters to the largely intergenic region upstream of rs498823 (see *Discussion*), we obtain a lower estimate of *s *=* *0.037, which is more in line with previous studies (Mathieson and Mathieson 2018; Stern *et al.* 2019).

## Discussion

We have shown how the effect of selective sweeps on nearby genealogies can be incorporated into the recursive description of the genealogical histories of a sample (Lohse *et al.* 2011). Much like a population bottleneck which can also be approximated as a multiple merger event (Bunnefeld *et al.* 2015), a selective sweep can be viewed as a discrete event that affects the genealogical history of a sample of neutral lineages (Kaplan *et al.* 1989). However, unlike bottlenecks, selective sweeps have a local effect on neutral variation in the genome (Galtier *et al.* 2000), lead to topologically unbalanced genealogies, and are therefore distinguishable.

While it is straightforward to recover previous analytic results for the expected loss of pairwise genetic diversity around sweep targets (Maynard Smith and Haigh 1974; Kaplan *et al.* 1989) and the SFS using the GF framework, our motivation was to extend analyses beyond expected coalescent times and pairwise samples. What we gain by embedding selective sweep approximations in the GF framework is a complete analytic description of the effects of genetic hitchhiking on the distribution of genealogies. Crucially, the strength and age of selective sweeps distort genealogies at nearby neutral sites in distinct ways. While these two aspects of past selective events are hard to disentangle from the expected reduction in genetic diversity, we show that they can be jointly estimated using richer summaries of sequence variation that capture information contained in the distribution of genealogies. Specifically, we show that for a single strong selective sweep, the bSFS has reasonable power to jointly infer both parameters even for a sample of *n *=* *4 lineages. Being able to maximize the information contained in small samples not only provides an obvious avenue for *CL* inference but also increases the power of comparative population genetic analyses, which are still limited by the lack of large resequence data sets for most taxa.

While our test on simulated data shows that, at least in principle, a sweep scan based on the bSFS has greater power than SweepFinder2, our exploration of the lactase sweep in humans illustrates that further work is required to apply such scans to real data. The fundamental difficulty is that our assumption that sequence variation is only ever indirectly affected by sweeps is at odds with the reality of selective constraints acting on coding and regulatory sequence. Thus in practice, justifying the assumption that blockwise variation around sweeps is selectively neutral and statistically exchangeable requires careful filtering decisions on the data. Alternatively, one can try and incorporate independent prior knowledge about selective constraint and mutation rate heterogeneity (Huber *et al.* 2016), *e.g.*, from background selection maps (Mcvicker *et al.* 2009) to model variation in *θ* among blocks.

### Model extensions and limitations

#### Star-like *vs* Yule

Throughout this paper, we have focused on two sweep approximations. While the instantaneous Yule approximation is a more accurate description of a hard sweep than the star-like approximation, we find very little difference in terms of power and accuracy between both sampling formulae in the case of a classic hard sweep. However, it may be unsurprising that ignoring the possibility of a family of early recombining lineages has little impact given that the (sub)sample size we considered is small (Pfaffelhuber *et al.* 2006).

#### Different types of selection

Although there has been much interest in differentiating the signatures of soft and hard sweeps (Hejase *et al.* 2020b), previous analytic work has shown that old hard sweeps are difficult to distinguish from soft sweeps given that both cause a partial reduction in genetic diversity (Hermisson and Pennings 2005; Pennings and Hermisson 2006a, 2006b). While recent soft sweeps can be distinguished by conspicuous patterns in haplotype data (Ferrer-Admetlla *et al.* 2014), these associations break down relatively quickly, and an old soft sweep may be indistinguishable from a (slightly older) hard sweep (Schrider *et al.* 2015; Zheng and Wiehe 2019). Despite this, machine-learning methods appear capable of classifying different histories of selection (Hejase *et al.* 2020b). By incorporating models of soft selective sweeps (Hermisson and Pfaffelhuber 2008) into the GF framework, it should be possible to identify the characteristics signatures of these selective processes in the branch length distributions and/or gene tree topologies.

We focus on the effects of a single hard sweep. An alternative is to capture the aggregate effects of positive selection on patterns of neutral diversity throughout the genome (Juric *et al.* 2016; Booker *et al.* 2017). While the signal of any particular sweep is inherently limited (given the stochasticity of both the coalescent and the trajectories of selected alleles), one would expect there to be much more information about positive selection when aggregating signatures across the genome. Given that, for mathematical convenience, our starting point has been to assume a model in which the waiting time to a sweep is exponentially distributed with rate *δ* (see equation 2), the current description also yields the recursion for the GF under a model of recurrent sweeps. However, in order to obtain results for a biologically plausible and general model of recurrent sweeps at uniformly distributed selective targets, one would have to integrate over both sweep locations and the distribution of fitness effects (Stephan *et al.* 1992).

#### Joint inference of demographic history and selection

The majority of theoretical results for selective sweeps to date have assumed that there is no population structure and that, with the exception of a focal sweep, the population is at equilibrium: the adaptive mutation arises *de novo* in an otherwise neutral panmictic population of constant size. In reality, of course, natural populations are not at equilibrium (Brandvain and Wright 2016) and it remains challenging to jointly infer past demography and selective events (Li *et al.* 2012). The most successful approaches to date extend the approximate diffusion model of Kimura (1955) to describe the population-level allele frequency spectrum under nonequilibrium dynamics. However, solving the diffusion equation can be difficult. Zivković and Stephan (2011) obtain analytic results for histories of varying population size, but in combination with positive selection, only numeric solutions are possible (Williamson *et al.* 2005), except for very simplistic demographic histories (Evans *et al.* 2007). Crucially, these predictions are primarily used to infer the effects of *direct* selection by comparing allele frequency spectra among different classes of mutations (*e.g.*, coding *vs* noncoding). While this approach can provide demographically explicit predictions for the background SFS in sweep-scanning methods (Pavlidis *et al.* 2013; Johri *et al.* 2020), results to-date are again limited to the SFS and to very recent sweeps (*T_a_* = 0).

Even simple changes in demography, *e.g.*, bottleneck in population size, strongly affect the power of sweep detection methods (Galtier *et al.* 2000; Jensen *et al.* 2005; Teshima *et al.* 2006; Stephan 2019). With the GF approach, however, it is possible to model complex and dynamic demographic histories. Because we treat the sweep as a discrete event, it too can be incorporated into general models of demography. Population structure adds further complications for detecting sweeps. For example, the VolcanoFinder method to infer adaptive introgression after secondary contact must assume complete lineage sorting, and as a consequence, its power to detect introgression sweeps is limited to highly divergent populations (Setter *et al.* 2020). The GF method fully accounts for the sorting of lineages, and in this context, would permit the inference of adaptive introgression even from a recently diverged donor population.

### Toward more powerful inference of selection

The motivation for our analytic work is to improve the ability to make inferences about selection. We have explored one possible approach, a *CL* framework based on the bSFS for estimating parameters of individuals sweeps in some detail. However, there are several other promising avenues for developing inference.

Our results for the effect of sweeps on genealogical branches may prove to be powerful in the context of recent methods that infer the ARG and/or tree sequences (with or without branch length information) from phased data, such as ARGweaver (Rasmussen *et al.* 2014), RENT+ (Mirzaei and Wu 2017), tsinfer (Kelleher *et al.* 2019), and RELATE (Speidel *et al.* 2019). In principle, the GF framework allows to connect a sequence of marginal trees inferred by these methods to explicit models of population structure and past selection.

One direction of further research could be to directly use the topology information contained in inferred tree sequences. This should also allow us to extend the calculation of the GF to larger sample sizes. Several summary statistics have been developed to diagnose the effect of sweeps on genealogical topologies (Li and Wiehe 2013; Yang *et al.* 2018). This research is motivated by the fact that statistics like root imbalance are invariant to population size changes. But, as far as as we are aware, results for the effect of sweeps on the distribution of topologies are lacking and could be used to improve sweep scans. For example, the probability of asymmetric topology (*i.e.*, a bipartition of {3, 1} in a sample of *n *=* *4) follows a nonmonotonic pattern around sweep targets. Analogous signals have been exploited to distinguish adaptive introgression sweeps from classic sweeps (Setter *et al.* 2020).

A final approach would be to compute the joint probabilities of the mutational configuration/branch lengths of a tree and its span. Leaving out the mutational information used to infer the tree sequences, inference would be based directly on the distribution of marginal genealogies, including the distribution of coalescence times (Weissman and Hallatschek 2017). While a full model of recombination, *i.e.*, allowing for an arbitrary number of recombination breakpoints in a sequence, seems infeasible, it should be possible to condition the GF on there being no recombination in a stretch of sequence of a given length. Abandoning the idea of nonrecombining blocks of a fixed length would thus allow us to incorporate LD information in the sweep inference. Although the direct inspection of the marginal trees that represent the genealogical history of a sample is an exciting prospect, we still require the statistical tools to exploit the information they contain about the evolutionary process efficiently.

## Data availability

The supporting figures as well as all notebooks and code used to generate and analyze the presented data can be found at https://github.com/GertjanBisschop/SweepsInTime.
